# Rejuvenation of the reconstitution potential and reversal of myeloid bias of aged HSCs upon pH treatment

**DOI:** 10.1111/acel.14324

**Published:** 2024-09-05

**Authors:** Sachin Kumar, Jeffrey D. Vassallo, Kalpana J. Nattamai, Aishlin Hassan, Angelika Vollmer, Rebekah Karns, Mehmet Sacma, Travis Nemkov, Angelo D'Alessandro, Hartmut Geiger

**Affiliations:** ^1^ Division of Experimental Hematology and Cancer Biology Cincinnati Children's Research Foundation Cincinnati Ohio USA; ^2^ Pharmacology Division CSIR‐Central Drug Research Institute Lucknow India; ^3^ Institute of Molecular Medicine Ulm University Ulm Germany; ^4^ Division of Gastroenterology, Hepatology and Nutrition Cincinnati Children's Hospital Medical Center and University of Cincinnati Cincinnati Ohio USA; ^5^ University of Colorado Denver—Anschutz Medical Campus Aurora Colorado USA; ^6^ Aging Research Center Ulm University Ulm Germany

**Keywords:** aging, hematopoietic stem cells, pH, polyamine, rejuvenation

## Abstract

Aged hematopoietic stem cells (HSCs) show reduced reconstitution potential, limiting their use in transplantation settings in the clinic. We demonstrate here that exposure of aged HSCs ex vivo to a pH of 6.9 instead of the commonly used pH of 7.4 results in enhanced HSCs potential that is consistent with rejuvenation, including attenuation of the myeloid bias of aged HSC and restoration of a youthful frequency of epigenetic polarity. Rejuvenation of aged HSCs by pH 6.9 is, at least in part, due to alterations in the polyamine/methionine pathway within pH 6.9 HSCs, and consequently, attenuation of the production of spermidine also attenuated aging of HSCs. Exposure of aged HSCs to pH 6.9, or pharmacological targeting of the polyamine pathway, might thus extend the use of HSCs from aged donors for therapeutic applications.

## Graphical abstract

Aged HSCs show compromised reconstitution potential and myeloid bias. We demonstrate that ex‐vivo exposure of aged HSCs to a pH of 6.9 enhances HSC potential and rejuvenation instead of the commonly used pH 7.4. Moreover, pH 6.9 attenuates the myeloid bias of aged HSC via regulating epigenetic polarity to young levels. These effects correspond to a decrease in the polyamine/methionine pathway of spermidine production and via pharmacological inhibitor, DFMO. This study suggests the prospect of using HSCs from aged donors for HSC transplantation therapy.

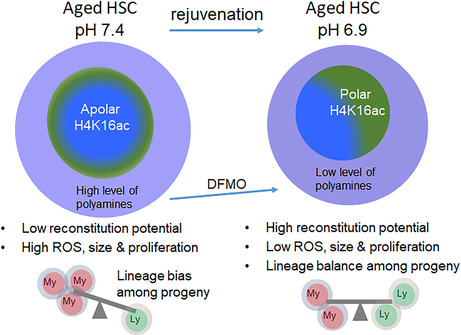

**Abbreviations**
BM: bone marrow
DFMO: D,L‐alpha‐difluoromethylornithine
H4K16ac: acetylated form of Histone 4 on lysine 16
HSCT: hematopoietic stem cells (HSCs), HSC transplantation therapy
LAMP2: chaperone‐mediated autophagy marker lysosome‐associated membrane protein 2
LC3: macro‐autophagy associated marker microtubule‐associated proteins 1A/1B light chain 3B
ODC: ornithine decarboxylase
pH: potential of hydrogen
pHi: intracellular pH
ROS: Reactive Oxygen Species
SMOX: spermine oxidase
SPN: spermidine
SRM: spermidine synthase

## 1 INTRODUCTION

Tissue attrition with age and thus aging is at least partly driven by the functional decline of stem cells (Rossi et al., 2008). In the hematopoietic system, the reconstitution and differentiation potential of hematopoietic stem cells (HSCs) declines with aging, which can lead to aging‐associated immune remodeling as well as contribute to aging‐associated myeloid neoplasms. Aging of HSCs, in general, is responsible for impaired aging‐associated hematopoiesis, including insufficient numbers of erythrocytes in the elderly (Geiger & Rudolph, 2009). Aged hematopoiesis in both mice and humans is further characterized by a differentiation skewing to myeloid lineages and a reduction of lymphoid output (Geiger et al., 2013). The aging of HSCs is driven by both cell‐extrinsic and cell‐intrinsic mechanisms. For example, aged HSC, when transplanted into young recipients, maintains to a great extent their age‐associated impaired hematopoiesis (Kumar & Geiger, 2017; Rossi et al., 2005). HSC transplantation therapy (HSCT) for bone marrow failures and leukemia conditions is therefore primarily performed with young donor cells in the allogeneic setting, while there is an age limit for autologous transplants (Copelan, 2006). This limits the number of patients that might benefit from HSCT. There is therefore a clinical demand for treatments that enhance the functional potential and attenuate the myeloid bias of aged HSC. On the molecular level, aging of HSCs is associated with, among others, changes in the level of activation of canonical Wnt signaling and changes in authophagy and metabolism (Chambers et al., 2007; Florian et al., 2012; Guidi et al., 2017; Kumar et al., 2021; Rossi et al., 2005). Aged HSCs also show a change in the distribution of polarity proteins in both the cytoplasm or the nucleus. For example, an apolar distribution of the epigenetic polarity protein histone 4 acetylated on lysine 16 (H4K16ac) is linked to an elevated level of asymmetric divisions of aged HSCs and myeloid skewing (Florian et al., 2018).

We recently described that a small change in pH, compared to the commonly used pH 7.4, maintains the function of HSCs from young animals upon short‐term ex‐vivo culture (Kumar et al., 2023). Here, we demonstrate that exposure of aged murine HSCs to a pH of 6.9 enhanced the reconstitution potential of aged HSC and restored a youthful level of myeloid contribution of the transplanted HSCs to the periphery. Furthermore, in addition to affecting growth, size, and autophagy, a pH of 6.9 also restored the frequency of cell polar for the distribution of acH4K16 in aged HSCs to the frequency reported for young HSCs. In aggregation, a change in pH rejuvenates central aspects of the function of aged HSCs. This finding suggests that changes in pH might help to extend the age range of HSC donors that qualify for HSCT applications.

## 2 RESULTS AND DISCUSSION

### 2.1 An ex vivo pH of 6.9 rejuvenates aged HSCs

Aged HSCs show distinct gene expression profiles, intracellular disorganization (aka apolarity of proteins), tend to preferentially differentiate into the myeloid lineage, and show reduced reconstitution potential upon serial transplantation (Mansell et al., 2023; Mejia‐Ramirez et al., 2020). The biology of aged HSCs is, therefore, distinct from the biology of young HSCs, and interventions that target young HSCs might work distinctly on aged HSCs. We recently reported a positive influence of a reduced pH (pH 6.9) for the temporary maintenance of young HSCs ex vivo (Kumar et al., 2023). Here, we determined whether levels in the ex‐vivo pH that are distinct from the commonly used pH of 7.4 affect the age of HSCs.

**FIGURE 1:**
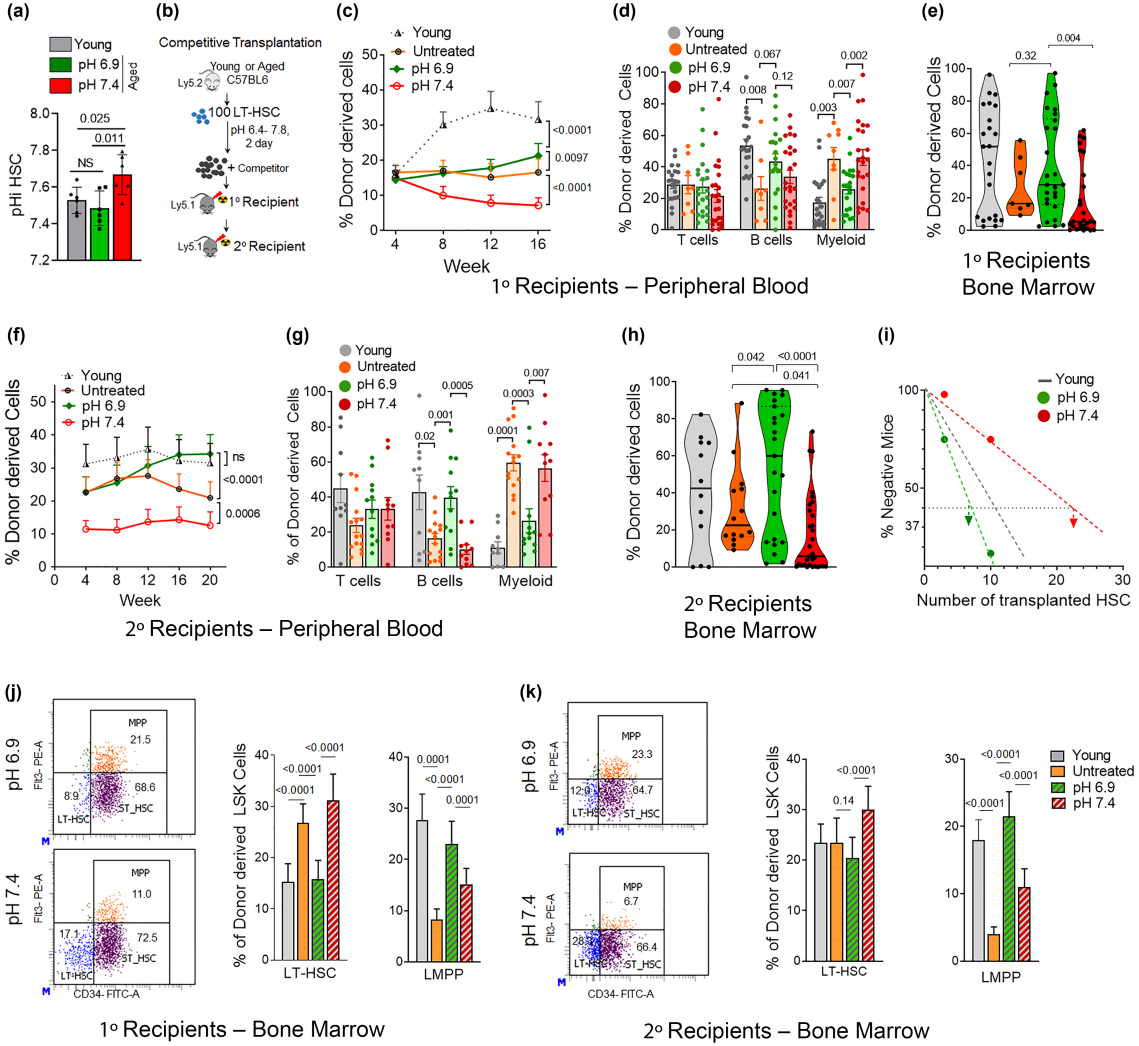
A pH of 6.9 in medium enhances aged HSC function. (a) Intracellular pH (pHi) of aged HSCs at an extracellular pH 7.4 or pH 6.9 or young HSCs at an extracellular pH of 7.4. *n* = 6 biological repeats, exact *p*‐values are mentioned between marked group; two‐tailed unpaired Student's *t*‐test. (b) Experimental setup of the competitive HSC transplants. HSC (100 cells) from C57BL/6 mice (Ly5.2) were exposed to pH 7.4 or pH 6.9 for 40 h and transplanted alongside 2 × 10^5^ cells from BoyJ mice (Ly5.1, competitor cells) into primary transplants, while for sary transplants 2 × 10^6^ BM cells from primary transplants were transplanted into irradiated BoyJ mice. (c, f) Contribution of the transplanted HSCs to donor‐derived Ly5.2+ cells in PB in primary (c) and secondary (f) recipients between 4 and 20 weeks post‐transplant. Exact *p*‐values are mentioned between marked groups; two‐tailed Student's *t*‐test. (d, g) Relative contribution of transplanted HSCs to T cells, B cells, and myeloid cells among donor‐derived Ly5.2+ cells in PB in primary (d) and secondary (g) recipients at 16 to 20 weeks post‐transplant. Exact p values are mentioned between marked group; two‐tailed unpaired Student's *t*‐test. (e, h) Relative Contribution of transplanted HSCs to donor‐derived Ly5.2+ cells to BM cells in primary (e) and secondary (h) recipients between 16 to 20 weeks post‐transplant. Exact *p*‐values are mentioned between marked group; two‐tailed Student's *t*‐test. (i) Limited dilution reconstitution analysis of aged HSC exposed to pH 7.4 or pH 6.9. Scored is any positive contribution of the low number of transplanted HSCs to donor‐derived Ly5.2+ cells in a competitive primary transplant setting. (j, k) Representative FACS dot plots depicting the gating strategy for LT‐HSC, ST‐HSC, and LMPP among LSK cells and the frequency of LT‐HSC and LMPP among donor‐derived LSK cells in primary (j) and secondary (k) recipients. Exact p values are mentioned between marked group; two‐tailed unpaired Student's *t*‐test. (c–k) *n* = 12–18 mice per group, based on 4–7 biological repeats of each experiment. Data from animals transplanted with untreated aged HSCs and young HSCs (exposed to pH 7.4) are represented by grey triangles or by grey in bar diagrams and violin plots. All data are mean ± SE except violin plots.

To this end, we first determined the intracellular pH (pHi) of aged HSCs. The pHi of aged HSCs was significantly elevated when compared to that of young HSCs (Figure 1a), consistent with the observation that the biology of aged HSCs is distinct from the biology of young HSCs. We next exposed aged HSCs for 1 h to an extracellular pH of 6.9 and compared the function of these cells to aged HSCs that were not cultivated at all and, young HSCs cultivated at the commonly used pH of 7.4. An extracellular pH of 6.9 in medium decreased pHi in aged HSCs to the pHi of young HSCs (pH 7.4) (Figure 1a), implying that changes in extracellular pH can restore pHi in aged HSCs to the level seen in young HSCs. We next tested the function of aged HSCs exposed to pHs ranging from 6.4 up to 7.8. One hundred purified (sorted) young (2–3 months old) and aged (20–22 months old) HSCs (Lin^−^Sca‐1^+^c‐Kit^+^CD34^−^Flt3^−^ cells) from BM were exposed to these distinct levels of pH for 40 h. Exposed HSCs were transplanted into primary and subsequently into secondary recipients (Figure 1b). Control recipients were transplanted with untreated fresh (non‐cultivated, NC) aged HSCs or young HSCs exposed to our standard pH of 7.4 (Figure 1b). A pH of 6.9 resulted in an optimum positive influence on chimerism in recipients supported by transplanted aged HSCs in the range of pHs (6.4–7.8) tested (Figure S1a,b). Aged HSCs exposed to pH 6.9, when transplanted, were superior in function to aged HSCs cultivated at pH 7.4 and supported an elevated level of chimerism in PB and BM in primary (Figure 1c,e) as well as secondary recipients (Figure 1f,h) in comparison to aged HSCs exposed to a pH of 7.4 or even fresh, uncultivated, aged HSCs. Even more encouraging, the level of reconstitution in the BM and PB of aged HSCs cultivated at pH 6.9 was similar to the level of reconstitution provided by young HSCs (pH 7.4) upon secondary transplantation (Figure 1f,h). Secondary transplants are the gold standard test for determining the long‐term function of HSCs. Limiting dilution experiments, in which HSCs were exposed to distinct levels of pH and subsequently transplanted at graded numbers into recipients to quantify HSC potential in more detail, confirmed this increase in the potential of aged HSCs exposed to pH 6.9 (Figure S1c), and up the to a level reported for young pH 7.4 HSCs (Figure 1i).

Upon aging, HSCs show myeloid skewing (more myeloid output, less lymphoid output), which is regarded as a central hallmark of aged HSCs (Florian et al., 2013). Aged pH 6.9 HSCs supported frequencies of myeloid cells (Figure 1d,g) and frequencies of B‐cells (Figure 1d,g) distinctly from the level supported by aged untreated or pH 7.4 exposed HSCs, but very similar to that found in animals reconstituted by young pH 7.4 HSCs (Figure S1d). Aged HSCs exposed to a pH of 6.9, upon transplantation, also showed levels of LT‐HSCs similar to that of young HSCs (pH 7.4), aka lower for young HSCs and aged pH 6.9 HSCs compared to aged untreated or aged pH 7.4 HSCs, in primary transplants (Figure 1j), and for LMPP (higher for young pH 7.4 HSCs) in both primary and secondary transplants (Figure 1j,k). Overall, a change in pH of the medium from 7.4 to 6.9 upon short‐term HSC ex vivo cultivation significantly enhanced the potential of aged HSCs and conferred rejuvenation of the function of aged HSCs for central aging parameters.

### 2.2 pH 6.9 restores epigenetic polarity to a youthful level

**FIGURE 2:**
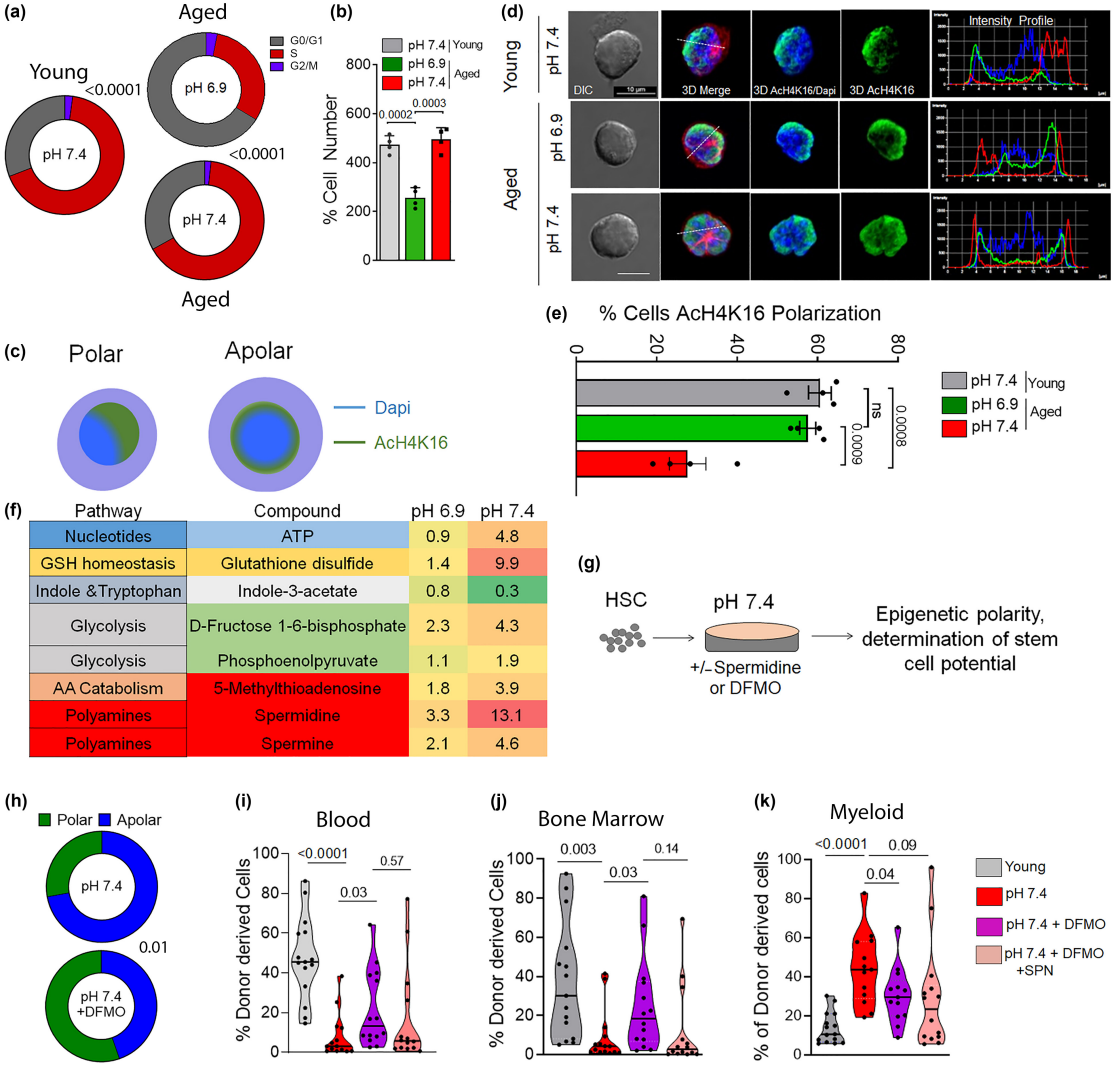
Changes in epigenetic polarity and low levels of polyamines are linked to the rejuvenation of HSCs by pH 6.9. (a) Percentage of cells in the G1, S and G2/M phase of the cell cycle upon exposure to pH 7.4 or pH 6.9. *n* = 4 biological replicates, exact *p*‐values are mentioned between marked group; two‐tailed unpaired Student's *t*‐test. (b) Percentage of the cell number post‐exposure/cultivation for 40 h relative to the total input of HSCs. *n* = 4 biological replicates, exact *p*‐values are mentioned between marked group; two‐tailed unpaired Student's *t*‐test. (c) Graphical representation of a polar and apolar distribution of proteins (Tubulin, AcH4K16) within an HSC. (d) Representative immunofluorescence pictures of pH 6.9 and pH 7.4 HSCs stained with anti‐AcH4K16 (green), anti‐tubulin (red), and nuclei stained with DAPI (blue), including the determination of an intensity profile through the cell. Pictures are shown as individual color images that are a 3D reconstruction of a Z‐stack capture and the overlay of color pictures and the overlay with DICs. Bar = 10 μm. (e) Percentage of pH 7.4 and pH 6.9 HSCs polarized for AcH4K16 compared to young controls. *n* = 4 biological replicates, exact *p*‐values are mentioned between marked group; two‐tailed unpaired Student's *t*‐test. (f) Table representing pathways and median changes in metabolites in HSCs at pH 6.9 and pH 7.4 relative to untreated (fresh) cells. *n* = 3 biological replicates. (g) Experimental set‐up for determining epigenetic polarity and reconstitution potential of HSCs upon modulation of the polyamine pathway by DFMO and DFMO/Spermidine (SPN) treatment. (h) Percentage of aged HSCs polarized for AcH4K16 in response to treatment with DFMO at pH 7.4, at least 50 cells per replicate, *n* = 2 biological replicates. Exact *p*‐values are mentioned between marked group; two‐tailed unpaired Student's *t*‐test). (i, j) Percentage of donor‐derived Ly5.2+ hematopoietic cells in peripheral blood (i) and BM (j) in animals transplanted with 100 HSCs (young or cultured at pH 7.4, in the presence of DFMO or in combination with SPN. Data in Figures (i, j) are from *n* = 12–16 recipient mice from two independent biological transplantation experiments. *p*‐values within the figure; two‐tailed unpaired Student's *t*‐test. (k) Contribution of myeloid cells among donor‐derived Ly5.2+ cells in PB. Exact *p*‐values are mentioned between marked group; two‐tailed unpaired Student's *t*‐test. All data are mean ± SE except violin plots.

We next determined cell growth, expansion dynamics, and cell metabolism upon exposure of aged HSCs to pH 6.9 or 7.4 or young HSCs. A lower frequency of cells in the cell cycle (Figure 2a) and thus only a 2‐fold expansion of aged HSCs was observed after 40 h at pH 6.9 compared to a 5‐fold expansion at pH 7.4 (Figure 2b). This lower cell cycle frequency at pH 6.9 was not associated with a drastic change in viability of cells, compared to pH 7.4 as well as young controls (Figure S2a). We also observed less intracellular reactive oxygen species (ROS) in aged pH 6.9 compared to pH 7.4 HSCs (Figure S2b). Lower levels of ROS are associated with a better function of HSCs (Ito et al., 2006; Jang & Sharkis, 2007). Aged pH 6.9 HSCs showed a decrease in cell size (determined by 3D confocal imaging, Figure S2c). They also showed a decreased volume of the nucleus (Figure S2d) compared to pH 7.4 HSCs, which was more similar to that of young HSCs, which is consistent with recent reports that smaller HSCs present with an enhanced potential (Kumar et al., 2023; Lengefeld et al., 2021) and that other interventions that result in rejuvenation of HSCs reduce the size of the nucleus similar to the size seen in young HSCs (Grigoryan et al., 2018).

We further observed an increase in the volume of chaperone‐mediated autophagy marker lysosome‐associated membrane protein 2 (LAMP2) in aged HSCs at pH 6.9 compared to pH 7.4, similar to young controls (Figure S2e,f), while the macro‐autophagy associated marker microtubule‐associated proteins 1A/1B light chain 3B (LC3) showed a decrease in aged pH 6.9 HSCs that was interestingly lower compared to that of aged pH 7.4 HSCs or young HSCs (Figure S2e,g). It is therefore a possibility that at a pH of 6.9, chaperone‐mediated lysosomal autophagy is increased in aged HSCs, concomitant with a decrease in macro‐autophagy. While elevated levels of LC3 autophagy are thought to be beneficial for the function of aged HSCs (Ho et al., 2017), also reduced levels of lysosomal activity/autophagy are linked to enhanced HSCs function (Liang et al., 2020). Additional research will be necessary to determine the level of dependency of aged HSCs on combinations of distinct forms of autophagy.

Epigenetic alterations in HSCs might likely link a long‐term change in the potential of aged HSCs upon transplantation to only a temporary ex vivo treatment at a pH of 6.9. Epigenetic changes are indeed tightly linked to HSC aging and can thus serve as valid biomarkers of rejuvenation (Beerman & Rossi, 2014; Guidi & Geiger, 2017). For example, repolarization of the nuclear distribution of H4K16ac has been reported to be tightly linked to the rejuvenation of the function of aged HSCs (Florian et al., 2012, 2018; Mejia‐Ramirez et al., 2020) (Figure 2c). In HeLa cells, a low pH has been reported to cause a global decrease and redistribution of the acetylated form of Histone 4 on lysine 16 (H4K16ac) (McBrian et al., 2013), while in general, the interplay between pH and epigenetics is not well studied in primary cells (McBrian et al., 2013). In contrast to aged HSC at pH 7.4, At pH 6.9, the frequency of aged HSCs with a polar nuclear distribution of H4K16ac was similar to that of young HSCs (Figure 2d,e). pH in itself is though not a general modifier of the level of histone acetylation in HSCs, as, for example, the localization of H3K27ac (a bona fide marker of activation of transcription) was not altered in pH 6.9 aged HSCs (Figure S2h,i). Interestingly, the overall levels of H3K27ac and H4K16ac were increased upon aging, by not affected by pH in aged HSCs (Figure S2j). In aggregation, the data suggest that a restoration of a youthful function of aged HSCs by exposition to a pH of 6.9 is linked to a low number of divisions and low cellular ROS together with changes in autophagy and a youthful level of rewiring of polarity of H4K16ac, all of which are phenotypic changes in aged HSCs that might contribute to rejuvenation of the function of aged HSCs upon exposure to pH 6.9. The identification of interconnections of causes and consequences among them will require additional investigations.

### 2.3 The polyamine pathway is critical for rejuvenation of aged HSC

We previously demonstrated that changes in the extracellular pH affect the metabolic wiring of young HSCs. We thus tested the extent to which a change in pH also influences the level of metabolites in aged HSCs (Reisz & D'Alessandro, 2017). Levels of ATP and glycolysis intermediates were reduced in aged pH 6.9 HSCs compared to aged pH 7.4 HSCs. Similarly, aged 7.4 HSCs showed a reduced level, relative aged 6.9 HSCs, of glutathione disulfide but also metabolites of the polyamine/methionine pathway that included spermine, spermidine, and 5‐Mehtylthioadenosine (Figure 2f, Table S1). The level of expression of gene products regulating the level of polyamines followed this lower trend (Table S2). Peroxisomal N(1)‐acetyl‐spermine/spermidine oxidase (PAOX) and Spermidine Synthase (SRM), polyamine biosynthesis was decreased, while Diamine acetyltransferase 1 (SAT1) and Spermine oxidase (SMOX), catabolic processes) was increased at pH 6.9 compared to pH 7.4 HSCs (Figure S2k). Aged 6.9 HSCs thus show lower levels of metabolites of the polyamine/methionine pathway compared to aged pH 7.4 HSCs, similar to the effect of changes in pH seen in young HSCs. A regulation of the level of polyamine metabolites in HSCs by pH is thus likely a general consequence of HSCs to the change in extracellular pH.

D,L‐alpha‐*difluoromethylornithine* (*DFMO*) inhibits ornithine decarboxylase (ODC) to decrease the levels of putrescine and spermidine (Kumar et al., 2023). Exposure of aged pH 7.4 HSCs to DFMO allows testing for causal links between changes in the polyamine pathway in aged HSCs and the rejuvenation of their function (Figure 2g). In the presence of DFMO (5 mM), aged 7.4 HSCs restored the aging bio‐marker epigenetic polarity, with now a primarily polar and thus youthful nuclear distribution of H4K16ac in aged HSCs (Figure 2h). As the polarity of H4K16ac is tightly associated with a long‐term youthful function of aged HSCs (Florian et al., 2018), the re‐polarization of this epigenetic markers by DFMO is thus likely linked to the long‐term effect of the short‐term pH treatment ex vivo. This implies that the polyamine pathway is critical novel regulator of aging‐associated changes in the epigenetics of HSCs. Upon DFMO treatment, there was also a reduction in cell volume (Figure S2l) in aged pH 7.4 HSCs, which rendered the size of these HSCs similar to pH 6.9 or young, pH 7.4 HSCs (Figure S2c). Addition of spermidine (SPN, 1 μM) muted the effect of DFMO on cell size of aged pH 7.4 HSCs (Figure S2l), validating spermidine as a target of DFMO. Experiments in which aged HSCs treated with DFMO or with DFMO and SPN were subsequently transplanted into recipients to determine their function revealed that animals transplanted with DFMO treated aged HSCs showed an elevated level of chimerism in blood as well as in BM, with a level in BM that was close to the level observed for young pH 7.4 HSCs (Figure 2i,j). Addition of SPN again blunted the response to DFMO, confirming the level of SPN to be a/the critical functional metabolite in aged HSCs that can confer aging of function in aged HSCs. Moreover, the myeloid bias of aged pH 7.4 HSCs shifted towards a lower and thus more youthful myeloid contribution in animals transplanted with DFMO‐treated aged pH 7.4 HSCs (Figure 2k), while this myeloid shift was only partially affected by the additional presence of SPN. Finally, we excluded that simply cell cycle attenuation, as observed for aged HSCs exposed to a pH of 6.9 (Figure 2a) results in re‐polarization and thus likely rejuvenation. Aged pH 7.4 HSCs, which were treated with the established CDK4/6 inhibitor Palbociclib (Deng et al., 2018; Purba et al., 2019) for 40 h, and which showed a lower number of cells upon expansion, similar to pH 6.9 (Figure S2m; Figure 2a,b), did not re‐polarize aged HSCs for H4K16ac distribution (Figure S2n; Figure 2e).

In summary, our data reveal that exposing aged HSCs short‐term (for approximately 2 days/40 h) to an extracellular pH of 6.9 results in the rejuvenation of the function of aged murine HSCs. DFMO, by muting the production of polyamines, phenocopies to a large extent the changes conferred on aged HSCs by pH 6.9. pH regulates epigenetic polarity and polyamine pathway metabolites in aged HSCs and controls by this means HSC phenotypes associated with aging. Targeting pH or pH‐regulated metabolites in aged HSCs rejuvenates the function of aged murine HSCs. Short‐term cultivation of HSCs ex vivo is necessary for genetic manipulations of HSCs or for processing of HSCs for subsequent transplantation. The precise cellular and molecular mechanisms that link HSC aging, metabolism, polyamines, and epigenetics need to be further investigated to allow for translation of these findings into clinical applications.

## 3 MATERIALS AND METHODS

### 3.1 Experimental animals

Young female C57BL/6J mice (2–3 months old), BoyJ mice (2–3 months old), and aged female C57BL/6 mice (20–24 months old) were obtained from the National Institute on Aging (NIA) mouse repository and were housed in mouse facility at Cincinnati Children's hospital medical center, Cincinnati, USA. All experimental procedures were approved by the Institutional Animal Committee at Cincinnati Children's hospital medical center, Cincinnati, USA.

### 3.2 Calibration of pH in medium and culture conditions

pH of Iscove‐modified Dulbecco medium (IMDM; Cellgro) was calibrated with 1 M HCl or 1 M NaOH to obtain the desired range upon overnight incubation at 37°C under 3% O_2_ and 5% CO_2_ settings, as described recently (Kumar et al., 2023). The pre‐calibrated media with distinct pH values were used for the culture of HSCs. Murine LT‐HSCs were cultured under 37°C, 5% CO_2_, 3% O_2_ conditions in IMDM containing 10% FBS & PS pre‐calibrated for distinct pH in the presence of cytokines (Flt‐3 L, SCF, and TPO at 50 ng/mL concentration) for 40 h. Half of the cultivation media was replaced every 24 h of culture with the medium of the same pre‐calibrated pH and containing all the cytokines and compounds like DFMO (5 mM) or SPN (1 μM) and CDK4/6 inhibitor, Palbociclib (1 μM). Details for Reagents and tools used in the present study are provided in Table 1.

**TABLE 1:** Reagents and tools table.

Reagent/resource	Reference or source	Identifier or catalog number
Experimental models		
C57BL/6J (*M. musculus*)	Jackson Lab	B6.129P2Gpr37tm1Dgen/J
BoyJ (CD45.1) mice	Jackson Lab	B6.SJL‐Ptprca Pepcb/BoyJ
Recombinant DNA		
‐		
Antibodies		
Biotin Mouse Lineage Panel	BD Biosciences	Cat # 559971
Streptavidin‐APC‐Cy7	BD Biosciences	Cat # 554063
Anti‐CD11b	eBioscience	clone M1/70
Anti‐B220	eBioscience	clone RA3‐6B2
Anti‐CD5	eBioscience	clone 53–7.3
Anti‐Gr‐1	eBioscience	clone RB6‐8C5
Anti‐Ter119	eBioscience	13–5921‐82
Anti‐CD8a	eBioscience	clone 53–6.7
Anti‐Sca‐1	eBioscience	clone D7
Anti‐c‐Kit	eBioscience	clone 2B8
Anti‐CD34	eBioscience	clone RAM34
Anti‐Flk2	eBioscience	clone A2F10
Streptavidin, AF 700	ThermoFisher	Cat # S21383
CD34‐FITC, clone RAM34	eBioscience	Cat # 11‐0341‐82
Flt‐3 PE	eBioscience	Cat # 12‐1351‐82
C‐KIT–APC	eBioscience	Cat # 17‐1171‐82
Sca‐1 PE‐Cy7	eBioscience	Cat # 25‐5981‐82
cKit–APC‐Cy7	eBioscience	Cat # A15423
CD45.2–FITC	Biolegend	Cat # 109806
CD45.1–PE	eBioscience	Cat # 12‐0453‐82
Anti‐CD3e–PE‐Cy7	Biolegend	Cat # 100320
Anti‐LAMP2 antibody, GL2A7	Abcam	Cat # ab13524
Anti LC3A/B	Cell Signalling	Cat # 4108
AcH3K27	Millipore	Cat # 07–360
AcH4K16	Abcam	Cat # ab13524
Anti‐rat dylight488‐conjugated antibody	Jackson ImmunoResearch	Cat # 112‐485‐071
Anti‐Rabbit Alexa Fluor 555	Invitrogen	Cat # A32794
Oligonucleotides and other sequence‐based reagents		
‐		
Chemicals, enzymes and other reagents		
96‐well round bottom plates (TPP Tissue culture plates)	Midwest Scientific	TP92097
96‐ well flat bottom plates	Suspension Sarstedt	Cat # 83.3924
Antifade Mounting Media	VECTASHIELD Laboratories	Cat # H‐1000‐10
Carboxy SNARF‐1 AM	ThermoFisher	Cat # C1272
Heat‐Inactivated Fetal Bovine Serum	GIBCO	Cat# 10438‐026
DCF‐DA	Invitrogen	Cat # D399
Click‐iT Plus EdU AF488 Assay Kit	Thermo Fisher	Cat # C10420
SMART‐Seq v4 Ultra Low Input RNA kit	Clontech	Catalog # 634892
Nextera XT DNA Library Preparation kit	Illumina	Catalog # FC‐131‐1096
Donkey Serum	Sigma	Cat # 566460
Iscove modified Dulbecco medium (IMDM	Cellgro,	Cat#21‐020‐CV
Penicillin/Streptomycin	Corning	Cat# 30‐002‐CI
Histopaque 1083	Sigma	Cat# 10831
DAPI	ThermoFisher	Cat # D1306
Nigericin	Sigma	Cat # N7143
NaCl	Sigma	Cat # S7653
KCl	Sigma	Cat # P5405
HCl	Sigma‐Merck	Cat # H1758
NaOH	Sigma	Cat # S8045
7AAD	Life Technologies	Cat # A1310
Cytokines		
Mouse stem cell factor	Prospec	CYT‐275
Murine thrombopoietin	Prospec	CYT‐346
Human recombinant G‐CSF	Amgen	Neupogen 300 μg/ml
Human Recombinant TPO	Stemcell Technologies	78,210
Human Recombinant IL‐3	Stemcell Technologies	78,040
Human Recombinant IL‐6	Stemcell Technologies	78,050
Human Recombinant SCF	Stemcell Technologies	78,062
Human Recombinant Flt3/Flk‐2 Ligand	Stemcell Technologies	78,009
Software		
GraphPad Prism8.0	https://www.graphpad.com	

### 3.3 HSC cell sorting and competitive transplantation

Low‐density bone marrow (LDBM) cells were isolated by density centrifugation (Histopaque 1083, Sigma) and stained with biotinylated lineage antibodies cocktail: anti‐CD11b (clone M1/70), anti‐B220 (clone RA3‐6B2), anti‐CD5 (clone 53–7.3) anti‐Gr‐1 (clone RB6‐8C5), anti‐Ter119 and anti‐CD8a (clone 53–6.7). After lineage depletion by magnetic separation (Dynabeads, Invitrogen), cells were stained with anti‐Sca‐1 (clone D7), anti‐c‐Kit (clone 2B8), anti‐CD34 (clone RAM34), anti‐Flk2 (clone A2F10), and Streptavidin antibodies, Stained LT‐HSCs (gated as Lin^−^Sca^+^Kit^+^CD34^−^Flk2^−^) were sorted in 96 well plates using a BD FACS Aria II (BD Bioscience) as previously reported (Florian et al., 2012; Kumar et al., 2023).

Murine LT‐HSCs (100 cells or as mentioned) from C57BL/6 mice (Ly5.2+) were cultured under diverse pH conditions or with pH 7.4 in the presence of DFMO (5 mM) or with SPN (1 μM) for 40 h in IMDM medium at 37°C (5% CO2, 3% O2). After incubation cells were harvested, washed twice. For transplantation, LT‐HSCs were mixed with 2 × 10^5^ BM cells from young (2‐ to 3‐month‐old) BoyJ competitor mice (Ly5.1+) and then transplanted into lethally irradiated young BoyJ recipient mice (Ly5.1+). The reconstitution potential was analyzed as donor‐derived chimerism in PB and BM at 4 to 20 weeks using immunostaining and analyzed on a CantoIII flow cytometer (BD Biosciences). Primary transplanted mice were sacrificed after 16–20 weeks, and BM chimerism was determined by FACS analysis. For secondary transplants, 3 × 10^6^ BM cells from an individual primary recipient mouse were injected into an individual secondary recipient BoyJ mouse. Serial BM transplantation experiments were repeated three to six times with a cohort of four or five recipient mice per donor. For the untreated group, sorted aged HSCs were transplanted without further incubation (Kumar et al., 2023), and young reference HSCs (exposed to pH 7.4) were also transplanted as a separate group.

### 3.4 Reconstitution analysis and donor‐derived chimerism

PB and BM cell immuno‐staining was performed according to standard procedures, and samples were analyzed on a CantoIII flow cytometer (BD Biosciences). Lineage FACS analysis data are plotted as the percentages of B220+, CD3+, and Myeloid (Gr‐1 + Mac‐1+) cells among donor‐derived Ly5.2+ cells, gated on viable cells based on FSC/SSC, following doublet discrimination. For hematopoiesis analysis, Lin‐ cells were then stained as aforementioned and analyzed using a CantoIII flow cytometer (BD Biosciences). LT‐HSCs and progenitors FACS analysis data were plotted as the percentage of LT‐HSC (gated as LSK CD34–/lowFlk2–), ST‐HSC (gated as LSK CD34 + Flk2–), and LMPP (gated as LSK CD34 + Flk2+) distribution among donor‐derived LSKs (Lin^_^c‐Kit+Sca‐1+ cells) (Florian et al., 2012). Primary transplanted mice were regarded as engrafted when PB chimerism was greater or equal to 1.0%, and contribution was detected in all lineages. Secondary transplanted mice were regarded as engrafted when PB chimerism was greater or equal to 0.5%, and contribution was detected in all three cell lineages.

### 3.5 Intracellular pH measurements

Measurement of pH_i_ for hematopoietic stem cells was performed using carboxy SNARF‐1 AM as described recently (Kumar et al., 2023). Briefly, intracellular pH was measured by both flow cytometry by incubating 2 × 10^6^ low‐density mononuclear cells stained for HSPC markers with a final concentration of 1 μmol/L *carboxy seminaphthorhodafluor*‐*1*‐*acetoxymethylester*, acetate (carboxy SNARF‐1 AM; Molecular Probes, Eugene, OR) for 20 min at 37°C. After washing with bicarbonate‐free buffer, PBS twice, SNARF labeled aliquots of cell suspensions were exposed to a specific pH, usually 6.8, 7.0, 7.2, 7.4, 7.6, and 7.8 containing high K^+^‐containing buffer (140 mM KCl, 1 mM MgCl_2_, 2 mM CaCl_2_, 5 mM glucose). Nigericin, H^+^/K^+^ antiporter (2 μg/mL) that abolishes the pH gradient across the cell membrane, was used for the generation of a calibration curve using a Canto III flow cytometer (Becton Dickinson). And the ratio of the SNARF‐1 emission wavelengths 640/580 nm was used to estimate the pH_i_ from a calibration curve using Prism 5 software. pH_i_ values of young and aged HSCs at pH 7.4 or aged HSCs exposed at pH 6.9 for 30 min were determined using HSC‐specific standard curve.

### 3.6 IF staining

LT‐HSCs (5000 cells) upon culture, were seeded on fibronectin‐coated glass coverslips for 3 h. Cells were fixed, permeabilized, and blocked with 10% Donkey Serum. Primary antibodies for AcH4K16 (07‐329, Millipore), AcH3K27 (Millipore, 07‐360), LAMP2 (GL2A7 Abcam ab13524) or LC3A/B (Cell Signaling Technologies #4108) were incubated overnight at 4°C, followed by incubation of secondary fluorescence conjugated antibody at room temperature for 1 h. Coverslips were mounted after DAPI staining. Nikon confocal microscope A1R system (Nikon) equipped with a 63× PH objective was used to image the samples. Cell volume was measured in NIS software. AcH4K16, LC3, or LAMP2 data were analyzed in Z stack‐derived 3D images of cells (Florian et al., 2012).

### 3.7 Cell size, reactive oxygen species, and proliferation analysis

LT‐HSCs (1000 cells) were cultured in U bottom 96 well plates with particular pH levels for 40 h. Cell size and volume were determined using flow cytometer and confocal analyses. Reactive oxygen species (ROS) were measured using DCF‐DA (5 μM, Invitrogen‐Molecular probes) on a CantoIII flow cytometer (BD Biosciences). Cell cycle and proliferation were analyzed using Click‐iT Plus EdU Alexa Fluor 488 Flow Cytometry Assay Kit (Thermo Fisher) as per manufacturer instructions. EdU (10 μM) was allowed to incorporate for 90 min at 37°C (5% CO_2_, 3% O_2_) conditions and analyzed post‐click reaction and nuclear staining (Kumar et al., 2023).

### 3.8 RNA‐Seq analysis

100 LT‐HSCs, treated with pH 6.9 or pH 7.4, were processed for transcriptome analysis. SMART‐Seq v4 Ultra Low Input RNA kit (Clontech) was used for cDNA synthesis and amplification. Libraries were prepared with Illumina's Nextera XT DNA Library Preparation kit as per manufacturer instruction. Sequencing was done in a Hi‐Seq 2500 under paired end 75 bp sequencing conditions. Log2 values were used to cluster all the DEGs in Cluster 3.0 using un‐centered correlation and the complete linkage method.

### 3.9 Metabolomics studies

Sorted 5 × 10^3^ HSCs were treated with pH 6.9 or pH 7.4 conditions for 40 h. Cells were washed twice with PBS, pooled and collected in a volume of 20 μl, and frozen in liquid nitrogen. Cells were resuspended in an ice‐cold organic solution for cell lysis and extraction of metabolites. The organic solvents were dried off and resuspended in an aqueous buffer for LC–MS, as previously described (Reisz & D'Alessandro, 2017).

### 3.10 Measurement of HSC death under culture conditions and attenuation of cell cycle

For the analysis of the frequency of cell death, cells were stained with Annexin V‐PE (BD Biosciences) and cell impermeable nuclear binding dye 7AAD (1 μg/mL). Cells positive for Annexin V+ but not for 7AAD are scored as apoptotic cells, while dead cells show both Annexin V+ and 7AAD+. For the attenuation of the cell cycle upon 40 h cultivation, cells were treated at the established dose of 1 μM with the CDK4/6 inhibitor Palbociclib (Sigma) (Deng et al., 2018; Purba et al., 2019).

### 3.11 Statistics

GraphPad prism 8.0 software was used for statistical analysis. The group sizes (*n*), including biological or technical replicates, specific statistical tests used to determine significance, and *p*‐values are provided in the figure legends. *p*‐values <0.05 were considered statistically significant. All Data presented are means ± SE. Scatter dot plots depict the mean with error bars representing standard error (s.e.).

## AUTHOR CONTRIBUTIONS

Sachin Kumar: conceptualization, data curation, formal analysis, investigation, methodology, project administration, supervision, visualization, writing—original draft preparation, writing—review and editing. Jeffrey D. Vassallo: conceptualization, methodology, investigation. Kalpana J. Nattamai: data curation, formal analysis, methodology, investigation, project administration, visualization. Aishlin Hassan: data curation, formal analysis, investigation, project administration, visualization, writing—review and editing. Anglika Vollmer: data curation, formal analysis, methodology, investigation, project administration, visualization. Rebekah Karns: data curation, formal analysis, software, visualization. Mehmet Sacma: investigation, formal analysis, data curation, methodology, visualization, formal analysis, software. Trevis Nemkov: investigation, methodology, formal analysis. Angelo D'Alessandro: data curation, methodology, formal analysis, resources. supervision. Hartmut Geiger: conceptualization, funding acquisition, methodology, resources, project administration, supervision, writing—original draft preparation, writing—review and editing.

## FUNDING INFORMATION

S.K acknowledges support from ECR/2022/001939 grant from Science and Engineering Research Board (SERB), India. This work was supported by the SFB 1506 Aging@Interfaces (DFG) and the FOR 2674 (DFG).

## CONFLICT OF INTEREST STATEMENT

The Authors declare no competing financial interests related to the publication of this study.

## Supporting information


Figure S1.

Figure S2.

Table S1.

Table S2.

## Data Availability

All raw data, including these of sequencing experiments as well as source tables are fully available and can be made accessible as required. Data will be made fully available upon publication in the corresponding databases.

## References

[acel14324-bib-0001] Beerman, I. , & Rossi, D. J. (2014). Epigenetic regulation of hematopoietic stem cell aging. Experimental Cell Research, 329(2), 192–199. 10.1016/j.yexcr.2014.09.013 25261778 PMC4250347

[acel14324-bib-0002] Chambers, S. M. , Shaw, C. A. , Gatza, C. , Fisk, C. J. , Donehower, L. A. , & Goodell, M. A. (2007). Aging hematopoietic stem cells decline in function and exhibit epigenetic dysregulation. PLoS Biology, 5(8), e201. 17676974 10.1371/journal.pbio.0050201 PMC1925137

[acel14324-bib-0003] Copelan, E. A. (2006). Hematopoietic stem‐cell transplantation. The New England Journal of Medicine, 354(17), 1813–1826. 10.1056/NEJMra052638 16641398

[acel14324-bib-0004] Deng, J. , Wang, E. S. , Jenkins, R. W. , Li, S. , Dries, R. , Yates, K. , Chhabra, S. , Huang, W. , Liu, H. , Aref, A. R. , Ivanova, E. , Paweletz, C. P. , Bowden, M. , Zhou, C. W. , Herter‐Sprie, G. S. , Sorrentino, J. A. , Bisi, J. E. , Lizotte, P. H. , Merlino, A. A. , … Wong, K. K. (2018). CDK4/6 inhibition augments antitumor immunity by enhancing T‐cell activation. Cancer Discovery, 8(2), 216–233. 10.1158/2159-8290.Cd-17-0915 29101163 PMC5809273

[acel14324-bib-0005] Florian, M. C. , Dorr, K. , Niebel, A. , Daria, D. , Schrezenmeier, H. , Rojewski, M. , Filippi, M. D. , Hasenberg, A. , Gunzer, M. , Scharffetter‐Kochanek, K. , Zheng, Y. , & Geiger, H. (2012). Cdc42 activity regulates hematopoietic stem cell aging and rejuvenation. Cell Stem Cell, 10(5), 520–530. 10.1016/j.stem.2012.04.007 22560076 PMC3348626

[acel14324-bib-0006] Florian, M. C. , Klose, M. , Sacma, M. , Jablanovic, J. , Knudson, L. , Nattamai, K. J. , Marka, G. , Vollmer, A. , Soller, K. , Sakk, V. , Cabezas‐Wallscheid, N. , Zheng, Y. , Mulaw, M. A. , Glauche, I. , & Geiger, H. (2018). Aging alters the epigenetic asymmetry of HSC division. PLoS Biology, 16(9), e2003389. 10.1371/journal.pbio.2003389 30235201 PMC6168157

[acel14324-bib-0007] Florian, M. C. , Nattamai, K. J. , Dorr, K. , Marka, G. , Uberle, B. , Vas, V. , Eckl, C. , Andrä, I. , Schiemann, M. , Oostendorp, R. A. , Scharffetter‐Kochanek, K. , Kestler, H. A. , Zheng, Y. , & Geiger, H. (2013). A canonical to non‐canonical Wnt signalling switch in haematopoietic stem‐cell ageing. Nature, 503(7476), 392–396. 10.1038/nature12631 24141946 PMC4078992

[acel14324-bib-0008] Geiger, H. , de Haan, G. , & Florian, M. C. (2013). The ageing haematopoietic stem cell compartment. Nature Reviews. Immunology, 13(5), 376–389. 10.1038/nri3433 23584423

[acel14324-bib-0009] Geiger, H. , & Rudolph, K. L. (2009). Aging in the lympho‐hematopoietic stem cell compartment. Trends in Immunology, 30(7), 360–365. 19540806 10.1016/j.it.2009.03.010

[acel14324-bib-0010] Grigoryan, A. , Guidi, N. , Senger, K. , Liehr, T. , Soller, K. , Marka, G. , Vollmer, A. , Markaki, Y. , Leonhardt, H. , Buske, C. , Lipka, D. B. , Plass, C. , Zheng, Y. , Mulaw, M. A. , Geiger, H. , & Florian, M. C. (2018). LaminA/C regulates epigenetic and chromatin architecture changes upon aging of hematopoietic stem cells. Genome Biology, 19(1), 189. 10.1186/s13059-018-1557-3 30404662 PMC6223039

[acel14324-bib-0011] Guidi, N. , & Geiger, H. (2017). Rejuvenation of aged hematopoietic stem cells. Seminars in Hematology, 54(1), 51–55. 10.1053/j.seminhematol.2016.10.005 28088989 PMC5244470

[acel14324-bib-0012] Guidi, N. , Sacma, M. , Standker, L. , Soller, K. , Marka, G. , Eiwen, K. , Weiss, J. M. , Kirchhoff, F. , Weil, T. , Cancelas, J. A. , Florian, M. C. , & Geiger, H. (2017). Osteopontin attenuates aging‐associated phenotypes of hematopoietic stem cells. The EMBO Journal, 36(10), 1463. 10.15252/embj.201796968 28507084 PMC5430211

[acel14324-bib-0013] Ho, T. T. , Warr, M. R. , Adelman, E. R. , Lansinger, O. M. , Flach, J. , Verovskaya, E. V. , Figueroa, M. E. , & Passegue, E. (2017). Autophagy maintains the metabolism and function of young and old stem cells. Nature, 543(7644), 205–210. 10.1038/nature21388 28241143 PMC5344718

[acel14324-bib-0014] Ito, K. , Hirao, A. , Arai, F. , Takubo, K. , Matsuoka, S. , Miyamoto, K. , Ohmura, M. , Naka, K. , Hosokawa, K. , Ikeda, Y. , & Suda, T. (2006). Reactive oxygen species act through p38 MAPK to limit the lifespan of hematopoietic stem cells. Nature Medicine, 12(4), 446–451. 10.1038/nm1388 16565722

[acel14324-bib-0015] Jang, Y. Y. , & Sharkis, S. J. (2007). A low level of reactive oxygen species selects for primitive hematopoietic stem cells that may reside in the low‐oxygenic niche. Blood, 110(8), 3056–3063. 10.1182/blood-2007-05-087759 17595331 PMC2018677

[acel14324-bib-0016] Kumar, S. , & Geiger, H. (2017). HSC niche biology and HSC expansion ex vivo. Trends in Molecular Medicine, 23(9), 799–819. 10.1016/j.molmed.2017.07.003 28801069 PMC5600322

[acel14324-bib-0017] Kumar, S. , Nattamai, K. J. , Hassan, A. , Amoah, A. , Karns, R. , Zhang, C. , Liang, Y. , Shimamura, A. , Florian, M. C. , Bissels, U. , Luevano, M. , Bosio, A. , Davies, S. M. , Mulaw, M. , Geiger, H. , & Myers, K. C. (2021). Repolarization of HSC attenuates HSCs failure in Shwachman‐diamond syndrome. Leukemia, 35(6), 1751–1762. 10.1038/s41375-020-01054-8 33077869 PMC11334678

[acel14324-bib-0018] Kumar, S. , Vassallo, J. D. , Nattamai, K. J. , Hassan, A. , Karns, R. , Vollmer, A. , Soller, K. , Sakk, V. , Sacma, M. , Nemkov, T. , D'Alessandro, A. , & Geiger, H. (2023). pH regulates hematopoietic stem cell potential via polyamines. EMBO Reports, 24(5), e55373. 10.15252/embr.202255373 36943011 PMC10157373

[acel14324-bib-0019] Lengefeld, J. , Cheng, C. W. , Maretich, P. , Blair, M. , Hagen, H. , McReynolds, M. R. , Sullivan, E. , Majors, K. , Roberts, C. , Kang, J. H. , Steiner, J. D. , Miettinen, T. P. , Manalis, S. R. , Antebi, A. , Morrison, S. J. , Lees, J. A. , Boyer, L. A. , Yilmaz, Ö. H. , & Amon, A. (2021). Cell size is a determinant of stem cell potential during aging. Science Advances, 7(46), eabk0271. 10.1126/sciadv.abk0271 34767451 PMC8589318

[acel14324-bib-0020] Liang, R. , Arif, T. , Kalmykova, S. , Kasianov, A. , Lin, M. , Menon, V. , Qiu, J. , Bernitz, J. M. , Moore, K. , Lin, F. , Benson, D. L. , Tzavaras, N. , Mahajan, M. , Papatsenko, D. , & Ghaffari, S. (2020). Restraining lysosomal activity preserves hematopoietic stem cell quiescence and potency. Cell Stem Cell, 26(3), 359–376 e357. 10.1016/j.stem.2020.01.013 32109377 PMC8075247

[acel14324-bib-0021] Mansell, E. , Lin, D. S. , Loughran, S. J. , Milsom, M. D. , & Trowbridge, J. J. (2023). New insight into the causes, consequences, and correction of hematopoietic stem cell aging. Experimental Hematology, 125–126, 1–5. 10.1016/j.exphem.2023.07.002 37433369

[acel14324-bib-0022] McBrian, M. A. , Behbahan, I. S. , Ferrari, R. , Su, T. , Huang, T. W. , Li, K. , Hong, C. S. , Christofk, H. R. , Vogelauer, M. , Seligson, D. B. , & Kurdistani, S. K. (2013). Histone acetylation regulates intracellular pH. Molecular Cell, 49(2), 310–321. 10.1016/j.molcel.2012.10.025 23201122 PMC3893119

[acel14324-bib-0023] Mejia‐Ramirez, E. , Geiger, H. , & Florian, M. C. (2020). Loss of epigenetic polarity is a hallmark of hematopoietic stem cell aging. Human Molecular Genetics, 29(R2), R248–R254. 10.1093/hmg/ddaa189 32821941

[acel14324-bib-0024] Purba, T. S. , Ng'andu, K. , Brunken, L. , Smart, E. , Mitchell, E. , Hassan, N. , O'Brien, A. , Mellor, C. , Jackson, J. , Shahmalak, A. , & Paus, R. (2019). CDK4/6 inhibition mitigates stem cell damage in a novel model for taxane‐induced alopecia. EMBO Molecular Medicine, 11(10), e11031. 10.15252/emmm.201911031 31512803 PMC6783643

[acel14324-bib-0025] Reisz, J. A. , & D'Alessandro, A. (2017). Measurement of metabolic fluxes using stable isotope tracers in whole animals and human patients. Current Opinion in Clinical Nutrition and Metabolic Care, 20(5), 366–374. 10.1097/MCO.0000000000000393 28768294 PMC5794022

[acel14324-bib-0026] Rossi, D. J. , Bryder, D. , Zahn, J. M. , Ahlenius, H. , Sonu, R. , Wagers, A. J. , & Weissman, I. L. (2005). Cell intrinsic alterations underlie hematopoietic stem cell aging. Proceedings of the National Academy of Sciences of the United States of America, 102(26), 9194–9199. 15967997 10.1073/pnas.0503280102 PMC1153718

[acel14324-bib-0027] Rossi, D. J. , Jamieson, C. H. , & Weissman, I. L. (2008). Stems cells and the pathways to aging and cancer. Cell, 132(4), 681–696. 18295583 10.1016/j.cell.2008.01.036

