# Supplementary figures and images for: Rejuvenation of the reconstitution potential and reversal of myeloid bias of aged HSCs upon pH treatment
Source: Aging Cell. 2024 Sep 5;23(10):e14324. doi: 10.1111/acel.14324 (PMC11464122; doi:10.1111/acel.14324)

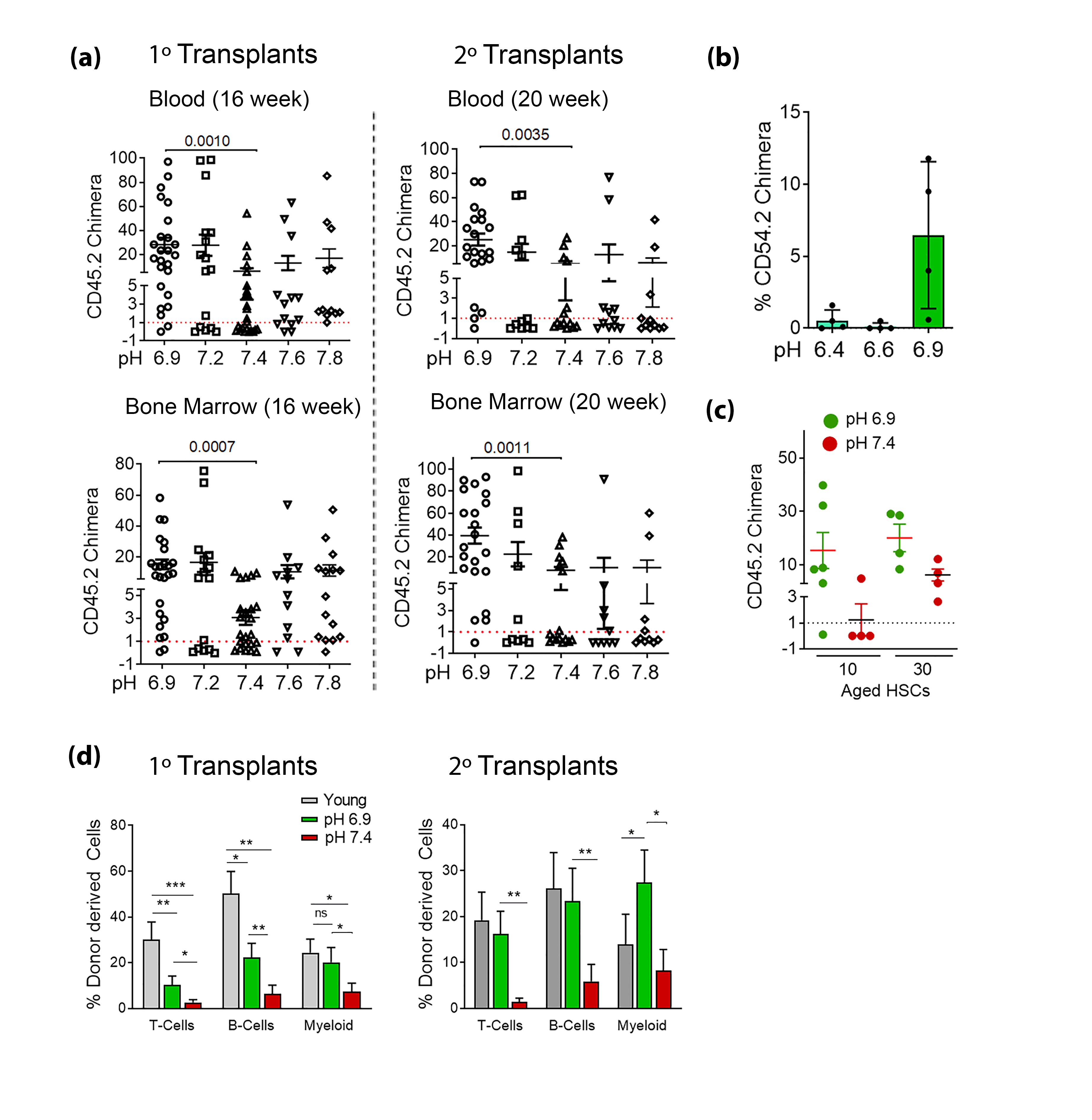

Supplement: Supplementary file 1 — Figure S1. [file ACEL-23-e14324-s001.tif]

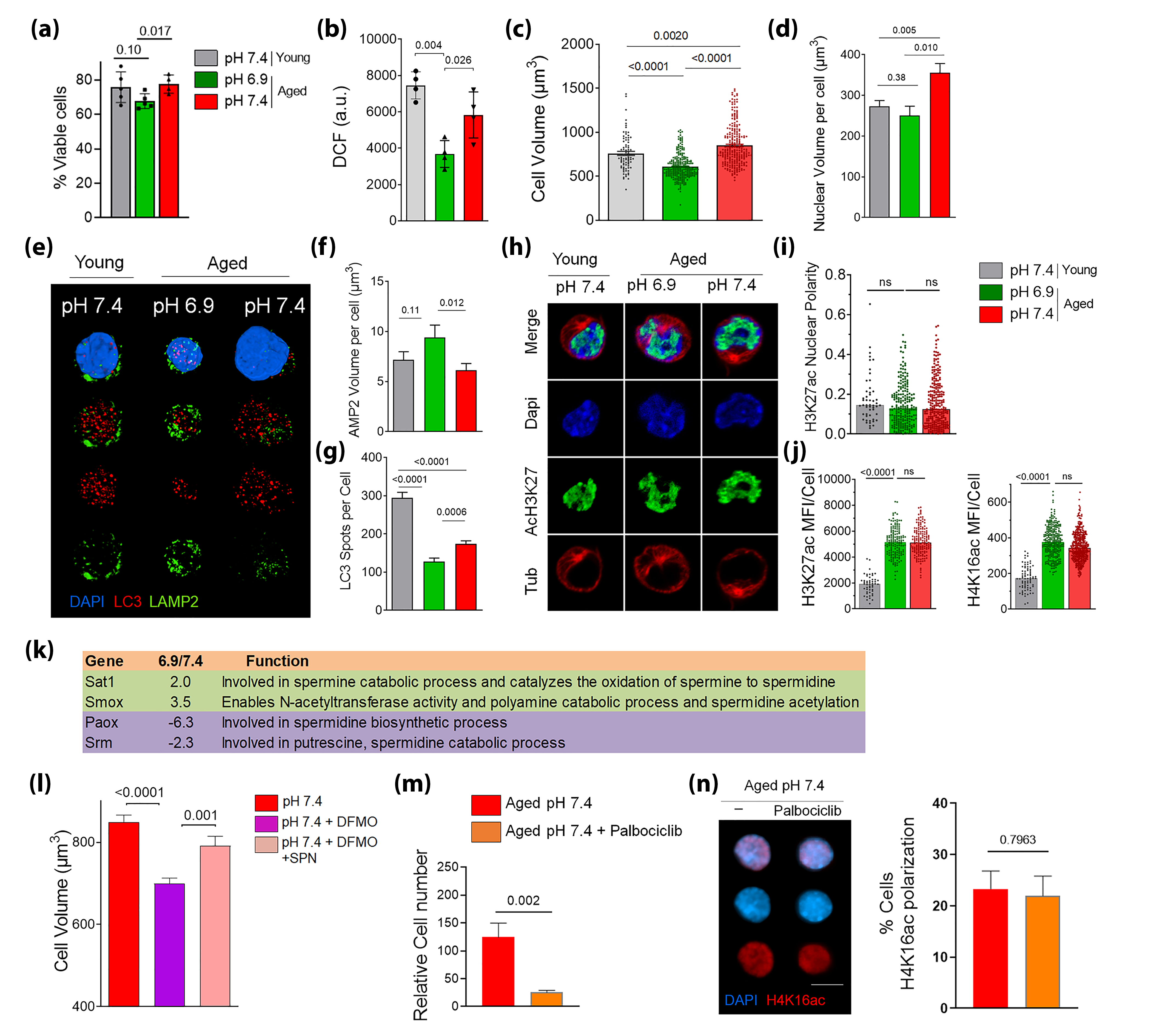

Supplement: Supplementary file 2 — Figure S2. [file ACEL-23-e14324-s004.tif]
